# Out‐of‐beam artifact suppression in charged nuclear fragment based carbon‐ion radiotherapy monitoring

**DOI:** 10.1002/mp.70351

**Published:** 2026-03-10

**Authors:** Rebekka Kirchgässner, Mária Martišíková, Patrice Schlegel, Pamela Ochoa‐Parra, Semi Harrabi, Oliver Jäkel, Jürgen Debus, Laurent Kelleter

**Affiliations:** ^1^ Heidelberg Institute for Radiation Oncology (HIRO) and National Center for Radiation Research in oncology (NCRO) Heidelberg Germany; ^2^ Division of Medical Physics in Radiation Oncology German Cancer Research Center (DKFZ) Heidelberg Germany; ^3^ Department of Physics and Astronomy Heidelberg University Heidelberg Germany; ^4^ National Center of Tumor Diseases (NCT) DKFZ and University Medical Center Heidelberg Germany; ^5^ Medical faculty Heidelberg University Heidelberg Germany; ^6^ Heidelberg Ion‐Beam Therapy Center (HIT) Department of Radiation Oncology Heidelberg University Hospital Heidelberg Germany; ^7^ Department of Radiation Oncology Heidelberg University Hospital Heidelberg Germany; ^8^ Clinical Cooperation Unit Radiation Oncology German Cancer Research Center (DKFZ) Heidelberg Germany

**Keywords:** carbon‐ion therapy monitoring, secondary ion tracking, Timepix3

## Abstract

**Background:**

Carbon‐ion radiotherapy offers highly precise targeting of tumors while sparing healthy tissue compared to X‐ray therapy. However, this precision comes at the cost of an increased sensitivity of the treatment to range uncertainties, which can arise from anatomical changes of the patient. Our group develops an in‐vivo treatment monitoring method by tracking of charged nuclear fragments using hybrid silicon pixel detectors.

**Purpose:**

Anatomical changes outside of the region accessed by carbon‐ion beams are clinically not relevant, as they do not affect the dose distribution. However, they can potentially influence the fragment data, producing artifacts, which might be interpreted as signals produced by clinically relevant anatomical changes. This misinterpretation would cause unnecessary clinical action, like performing a CT scan. This work proposes methods for the identification and suppression of clinically irrelevant artifacts with the aim of avoiding unnecessary clinical action.

**Methods:**

A clinically relevant and an irrelevant anatomical change are emulated by introducing coin‐sized air cavities at different positions in a homogeneous cylindrical plastic head phantom. Charged nuclear fragments are detected by a Timepix3‐based mini‐tracker during irradiations of this phantom with a clinically realistic treatment plan. All measurements are performed for two different positions of the mini‐tracker. The reconstructed fragmentation vertex distributions are analyzed and compared to those of reference measurements.

**Results:**

A significant signal from the clinically irrelevant air cavity was observed. This artifact was found to differ from the signal of the clinically relevant cavity. Most importantly, the location of the artifact changes with the mini‐tracker position, whereas the relevant signal remains unchanged. This facilitates identification of the artifact as well as its suppression by combining the data from several mini‐trackers at different positions around the patient.

**Conclusions:**

Clinically irrelevant changes were shown to potentially impede carbon‐ion treatment monitoring by tracking of charged nuclear fragments. However, positioning several mini‐trackers around the patient, which monitor the treatment from different perspectives, was found to be the key to the identification and suppression of artifacts from anatomical changes outside of the region accessed by carbon‐ion beams. This is implemented in the detection system of an ongoing clinical trial.

## 1 INTRODUCTION

Radiotherapy is among the most important treatment techniques for cancer, one of the leading causes of death worldwide.^1^ Radio‐resistant and deep‐seated tumors pose a special challenge and call for alternative modalities beyond the conventional X‐ray therapy. Carbon‐ion radiotherapy is such an alternative, demonstrating its advantages since the first clinical trials 30 years ago, especially but not only for head and neck cancer.^2^, ^3^ Firstly, with ions such as carbon ions or protons, the dose distribution can be shaped more precisely to the tumor than with X‐rays, thereby reducing the dose to surrounding healthy tissue. This is possible due to the finite range of the ions and their property of depositing most of their energy at the end of their path. This advantage is even stronger for carbon ions as compared to protons, due to reduced lateral scattering and range straggling. Secondly, carbon‐ions are biologically more effective than X‐rays and protons.^4^

The high precision of the dose distribution however, comes at the cost of a high sensitivity to uncertainties in the range of the carbon ions, which can among other factors arise from potential anatomical changes between treatment fractions. If such a change occurs in the path of the carbon ions, their range can be affected, which alters the dose distribution. If internal anatomical changes remain undetected in the clinical setting, they may lead to an underdosage of the tumor or overdosage of healthy tissue. In the case of head and neck tumors, which are the current focus of our group, internal changes might arise from the filling or emptying of nasal and oral cavities, tissue swelling or tumor growth or shrinkage. In the current clinical practice, this challenge is addressed by irradiating safety margins around the tumor to avoid underdosage. Additionally, some patients receive control CTs on some days during the treatment course depending on their individual risk of developing relevant anatomical changes. Daily CTs for all patients are not feasible due to the applied dose as well as hardware and personnel costs. Therefore, both actions do not provide daily feedback and increase the dose to healthy tissue.

Several monitoring methods, which could potentially offer daily feedback on internal changes without applying additional dose, are under investigation.^5^ They use secondary and tertiary radiation, which is produced during treatment by the interaction of the carbon ions with the patient's tissue. An effective monitoring method could give information on when a new CT for treatment plan adaptation is needed, thereby reducing the number of unnecessary CTs. It could also facilitate the reduction of safety margins which would reduce the amount of healthy tissue in the high dose region. Our group is working on one such monitoring method where charged nuclear fragments are tracked with the aim of detecting interfractional anatomical changes. This has proven promising in earlier studies: in a first patient trial at the Centro Nazionale di Adroterapia Oncologica (CNAO) in Italy an emptying of the nasal cavity in the path of the carbon ions could be detected in one of the patients.^6^

Our group uses mini‐trackers based on hybrid silicon pixel detectors to track charged nuclear fragments. The measured tracks are used to reconstruct a distribution of fragmentation vertices (FV distribution). When two such distributions from different treatment fractions are compared, anatomical changes can show as differences. This has previously been demonstrated using a homogeneous cylindrical Polymethyl Methacrylate (PMMA) head phantom with air gaps introduced at different depths.^7^ Furthermore, a deep seated change in an anthropomorphic head phantom was successfully detected and results were confirmed by MC simulations.^8^, ^9^ As a result, a clinical trial using a detection system with 28 Timepix3 detectors^10^ is currently ongoing at the Heidelberg Ion Beam Therapy Center (HIT, Heidelberg, Germany).

Anatomical changes of the patient affect the production, absorption and scattering of fragments, which leads to changes in the measured FV distributions. The production of fragments is mainly affected if the change occurs in the path of the primary carbon‐ions and therefore alters the range of the primary ions and the dose distribution. However, fragment scattering and absorption are also influenced by changes outside of the region accessed by primary carbon‐ion beams, which do not change the dose distribution in the tumor and are therefore of no clinical relevance. Signals arising from such out‐of‐beam changes would be considered artifacts. As such, they would pose a problem as they could lead to unnecessary additional CTs of the patient even though no plan adaptation is required. It is therefore vital to be able to classify anatomical changes as being located either in the beam or out of the beam.

The influence of material that is outside of the carbon‐ion beams but in the fragment path on measured FV distributions has previously been studied. It was found that the shape of a FV distribution from a single pencil beam depends on the amount of material between the beam and the tracker.^11^ This distortion gets more complicated when the material is heterogeneous^12^ as is the case in patients. A correction of the distribution based on prior knowledge of the patient's anatomy from a planning CT could remove the out‐of‐beam absorption and scattering effects. This approach is very challenging with respect to reliability and computational power, and it cannot take into account interfractional changes either, as daily control CTs are usually not available. Therefore, this correction does not remove artifacts caused by clinically irrelevant changes.

This work systematically studies, for the first time, the effect of interfractional changes outside of the carbon‐ion beams on monitoring using charged nuclear fragments. The signature of artifacts is compared to that of clinically relevant signals and a suppression method for the former is proposed.

## 2 MATERIALS AND METHODS

### 2.1 HIT

The experiments presented in this work were carried out at the HIT. At this facility, cancer patients are treated with protons, helium ions and carbon ions, all of which are also available for research purposes. Highly conformal dose distributions are achieved with the active scanning technique.^13^ Thereby, a narrow ion beam called pencil beam is scanned across the target volume. Its penetration depth is determined by the ion energy delivered by the accelerator whereas the lateral position is changed by two dipole magnets.

The Beam Application and Monitoring System (BAMS)^14^ measures important beam parameters online during irradiations. For each irradiation of a treatment plan, a beam record file is made available, which contains the measured lateral pencil beam positions with their respective timestamps, among other information.

### 2.2 Head phantom

**FIGURE 1:**
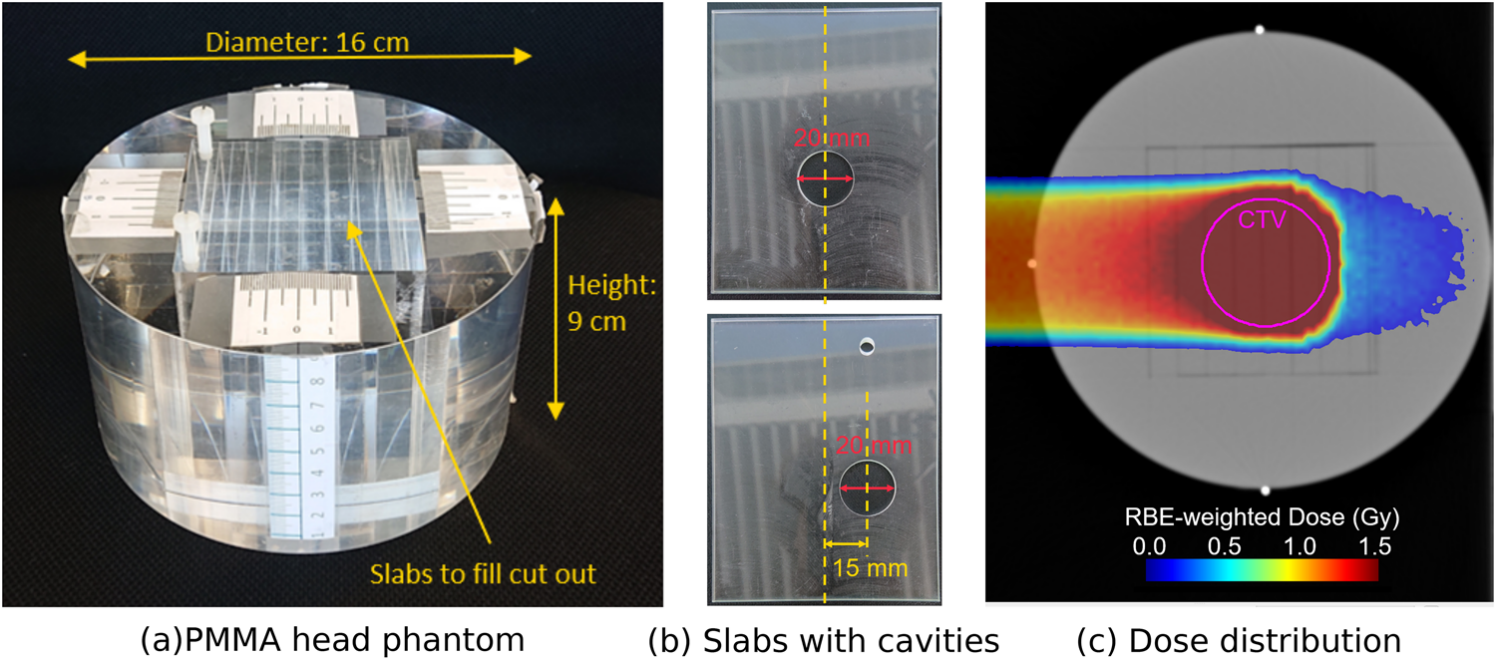
(a) Photo of the cylindrical PMMA head phantom with a $8\times 8\times 8\;{\mathrm{cm}}^{3}$ cut‐out filled with PMMA slabs. (b) PMMA slabs of 4 mm thickness with cavities. Top: in‐beam cavity. Bottom: out‐of‐beam cavity. (c) CT slice of the PMMA head phantom with the dose distribution of the field which was used for the measurements in this work. The virtual tumor (CTV) is indicated in pink.

For this work, a head‐sized PMMA cylinder was used as a head phantom. As shown in Figure 1a, a $8\times 8\times 8\;{\mathrm{cm}}^{3}$ cuboid is cut out of the cylinder. This cut‐out is filled with PMMA slabs to retain a homogeneous cylinder. Slabs with cylindrical holes of different adiameter and thickness are available to introduce air‐filled cavities into the phantom.

**FIGURE 2:**
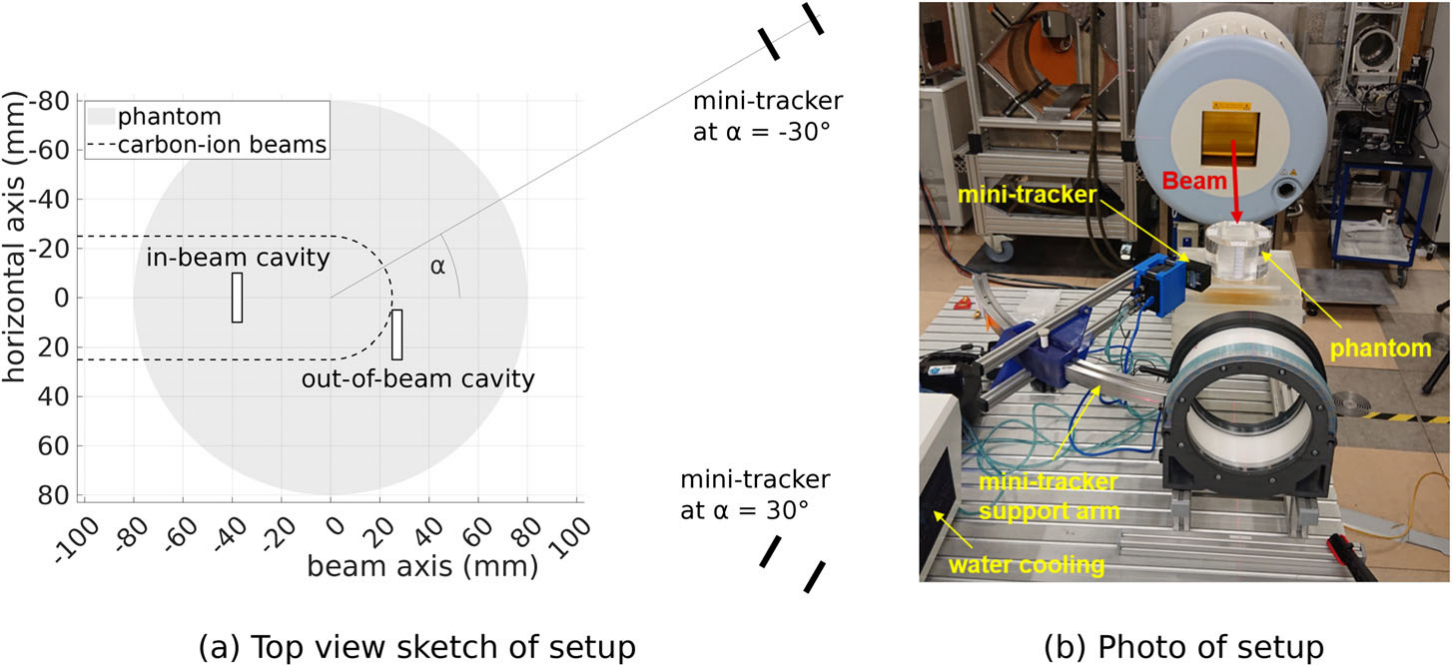
(a) Sketch of the experimental setup in top down view. All used cavity and mini‐tracker positions are displayed. (b) Photo of the setup in the experimental room at HIT. Here, the mini‐tracker is positioned at angle of $\alpha =30^{\circ }$ to the beam axis.

In this work, two such slabs with a cylindrical cavity of 4 mm thickness and 20 mm diameter were used, which are shown in Figure 1b. Laterally to the beam, the cavity shown in the top panel is located at the center of the treatment plan. Along the beam axis its upstream end is positioned 40 mm upstream of the isocenter. At this position, the cavity lies within the region crossed by the primary carbon ions and is thus called *in‐beam cavity* in the following. The cavity shown in the bottom panel is situated 15 mm to the right of the central position along the horizontal axis. Its upstream end is positioned 25 mm downstream of the isocenter. As a result, no primary carbon ions reach this second cavity, which is therefore called *out‐of‐beam cavity* in the following. The positions of the two cavities are depicted in Figure 2a with respect to the primary carbon‐ion beams and the phantom.

For the irradiation of the head phantom, a clinically realistic treatment plan was created with the syngo RT Planning system (Siemens Healthcare GmbH, Erlangen, Germany) based on a CT acquired with a clinical CT scanner (SIEMENS Sensation Open device). A virtual spherical tumor with a volume of 49.93 mL at the center of the head phantom is targeted with two equally‐weighted opposing fields, similar to clinical routine at HIT. The prescribed RBE‐weighted dose of this treatment is 60 Gy in 20 fractions, resulting in an RBE‐weighted fraction dose of 3 Gy. For the experiments presented here, only one of the two fields was used in order to save time. The dose distribution of this field is shown in Figure 1c.

### 2.3 Experimental setup

A mini‐tracker based on hybrid silicon pixel detectors (Advacam s.r.o., Prague, Czech Republic)^15^ was used in this work to track charged nuclear fragments originating from nuclear interactions of the clinical carbon‐ion beam with the head phantom. This mini‐tracker contains four Timepix3 chips^16^ arranged in two layers with a distance of 20.3 mm. In each layer, two Timepix3 chips share a silicon sensor of 500 μm thickness, resulting in a sensitive area of approximately $2.8\times 1.4\;{\mathrm{cm}}^{2}$. The pixel size of $55\times 55\;\mu m^{2}$ ensures precise spatial information. The Timepix3 technology allows for data driven readout of the arrival time of a signal with a binning of 1.5625 ns as well as the time over threshold, which serves as a measure of the deposited energy. In this work, the mini‐tracker was operated at a bias voltage of 200 V to ensure full depletion of the sensor. The detection threshold was set to 10 keV, which suppresses electronic noise to practically zero.

For the measurements, the center of the head‐sized PMMA phantom was aligned with the room isocenter using the in‐room laser system. The mini‐tracker was mounted on a support arm, which was also aligned using the in‐room laser system. This support arm facilitated the positioning of the mini‐tracker downstream of the head phantom at a distance of 20.7 cm to the isocenter as is visible in Figure 2. Thereby, the mini‐tracker was positioned at an angle α to the beam axis, meaning that the connection line between the centers of the two detection layers intersects the beam axis at the isocenter forming an angle α. In this work, two angles were used: $\alpha =30^{\circ }$ and $\alpha =-30^{\circ }$, resulting in equivalent fragment count numbers and spatial resolution for both mini‐tracker positions because of the axial symmetry. The absolute value of $30^{\circ }$ was found to be a suitable compromise between statistics and spatial resolution.^17^ The distance to the isocenter is similar as in the detection system for the ongoing clinical trial,^10^ where the detectors are placed as close to the patient as possible without risking collisions. Both mini‐tracker positions are indicated to scale in Figure 2a. The mini‐tracker was actively water‐cooled to stabilize the sensor temperature at around 30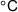.

### 2.4 Experimental procedure

To acquire the data presented in this work, the head phantom was irradiated with the treatment plan presented in Section 2.2 and simultaneously, tracks of charged nuclear fragments were recorded with the mini‐tracker. Like this, five measurements were conducted for each mini‐tracker position. The measurements differ by their configuration of the air cavities in the phantom, which are presented in Table 1. Only the first two measurements exhibit the same cavity configuration for the purpose of studying statistical fluctuations. The locations of the out‐of‐beam and the in‐beam cavity are described in Section 2.2. The resulting ten measurements each consisted of four irradiations of the treatment plan without any changes to the setup in between. These four irradiation repetitions were chosen as a compromise between emulating the detection system with seven mini‐trackers used in the ongoing clinical trial^10^ and the limited beam time. In the following, each measurement is always the sum of these four irradiations.

**TABLE 1:** Cavity configurations of the five measurements that were conducted for each mini‐tracker position.

Measurement No.	Out‐of‐beam cavity	In‐beam cavity
1	not present	not present
2 (for statistical study)	not present	not present
3	present	not present
4	not present	present
5	present	present

### 2.5 Data post‐processing

The raw data that is read out from the detector consists of the arrival time and the time over threshold of pixel signals. Several steps of data post‐processing are performed with a Matlab 2021 (MathWorks, Natick, MA, USA) code developed by this group to obtain the approximate fragmentation vertices from the raw data. Firstly, the time over threshold is converted to the deposited energy with a pixel‐wise calibration provided by Advacam. Secondly, neighboring pixel signals with similar arrival times need to be grouped into clusters due to charge sharing effects. Such a cluster represents a signal from a charged nuclear fragment, with the earliest arrival time within the cluster serving as the timestamp and the energy deposition weighted mean of the pixel locations as the cluster's position. Tracks are formed by finding coincident clusters in the front and back layer with a maximum absolute timestamp difference of 75 ns. If several clusters fulfill this criterion, the clusters with the smallest timestamp difference are paired. The measured fragment track is formed by drawing a straight line through the two cluster positions. Next, the timeline of the measured fragments is synchronized with the beam record file from the BAMS, thus assigning each fragment to the pencil beam it originated from. This facilitates the approximation of the fragmentation vertices in 3D by finding the shortest connection line between the measured track projected into the patient and the corresponding pencil beam. The fragment origin is then approximated at the center of this connection line. The data post‐processing results in a 3D distribution of estimated fragmentation vertices, which is the basis for the analysis presented below and is here abbreviated to *FV distribution*.

### 2.6 Analysis

The analysis in this work focuses on comparing a measured FV distribution to a reference distribution with the aim of finding significant differences between them, which could originate from the air cavities inserted into the phantom. The distributions are integrated along the vertical axis and all figures represent a top down view onto the phantom.

The comparison between the two FV distributions is done with a moving‐window analysis in the horizontal plane, meaning that for each pixel in a plot, all reconstructed fragmentation vertices within a window of 10×10 mm around the pixel center are used. The pixel centers form a 6×6 mm grid, which leads to a slight statistical dependence between neighboring pixels, as their windows overlap. Windows with less than 100 FVs are excluded from the analysis. These parameters offer a sufficient spatial resolution while at the same time ensuring enough statistics within each window.

The pixel values represent the difference between the two FV distributions in units of standard deviations D(σ), which is calculated by dividing the fragmentation vertex count difference by its standard deviation:

(1) $$D\left(\sigma \right)=\frac{\Delta N}{\sigma_{\Delta N}}=\frac{N_{\mathrm{meas}}-N_{\mathrm{ref}}}{\sqrt{N_{\mathrm{meas}}+N_{\mathrm{ref}}}}.$$

The standard deviation $\sigma_{\Delta N}$ is obtained from a Gaussian error propagation with Poisson errors for the numbers of fragmentation vertices $N_{\mathrm{meas}}$ and $N_{\mathrm{ref}}$.

## 3 RESULTS

### 3.1 Fragmentation vertex distribution and statistical fluctuations

**FIGURE 3:**
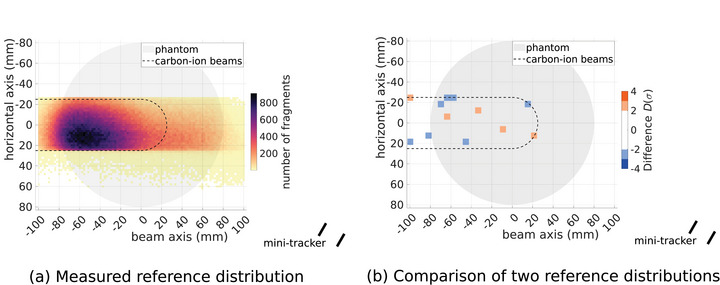
(a) Reference FV distribution without air cavities and the mini‐tracker at $\alpha =30^{\circ }$. The shaded area represents the phantom. The region passed by primary carbon ions is delineated with a dashed line. (b) Difference in units of standard deviation D(σ) between two measured FV distributions with identical setup as in (a).

Figure 3a shows a FV distribution in top down view measured for the homogeneous phantom without any air cavities and with the mini‐tracker at an angle of $\alpha =30^{\circ }$ to the beam axis. Along the beam axis, the maximum FV count is observed in the entrance region of the carbon‐ion beams. This is due to the high energy of the fragments created there, which decreases their probability of being absorbed in the phantom. Additionally, the further upstream a fragment is created, the smaller the angle to the beam axis at which it reaches the mini‐tracker. Most fragments are created with a small angle to the beam axis.^18^ Beyond the range of the primary carbon ions, only a small number of FVs is detected. This extended tail is caused by higher‐order fragments. Laterally, the distribution is concentrated on the region covered by the primary carbon ions, with a small bias towards the side of the mini‐tracker. Firstly, this is due to the reduced absorption and scattering probability for fragments created there, as they cross less material on their way to the mini‐tracker, as well as the smaller angle to the beam axis needed to reach the mini‐tracker. Additionally, the algorithm used for projecting the measured tracks does not allow for reconstructed FVs behind the corresponding pencil beam from the perspective of the mini‐tracker.^7^ This leads to the sharp cutoff of the distribution at −26 mm on the horizontal axis.

The distribution presented in Figure 3a acts as a reference for all measurements with the mini‐tracker at $\alpha =30^{\circ }$. A second reference distribution for the mini‐tracker position at $\alpha =-30^{\circ }$ was acquired, which shows the same characteristics as Figure 3a but flipped along the beam axis due to the symmetry of the setup. The results of this work are based on comparisons of measured distributions to the respective reference distribution with the same mini‐tracker position. Figure 3b presents the comparison of a second measurement without cavities to the reference. The experimental setup was not altered in any way between the measurements. Therefore, any observed differences can only originate from statistical fluctuations of the beam delivery as well as the fragment production and detection.

The differences observed in Figure 3b are compatible with statistical fluctuations. Of the 356 regions which are evaluated, because they contain at least 100 FVs, 12 show deviations above 2σ. This is in agreement with the expectation of 16 pixels passing the threshold of 2σ (4.55% of events) by chance based on normally‐distributed data. This demonstrates the absence of a signal in the absence of a density change in the phantom.

### 3.2 Density change in the fragment path

**FIGURE 4:**
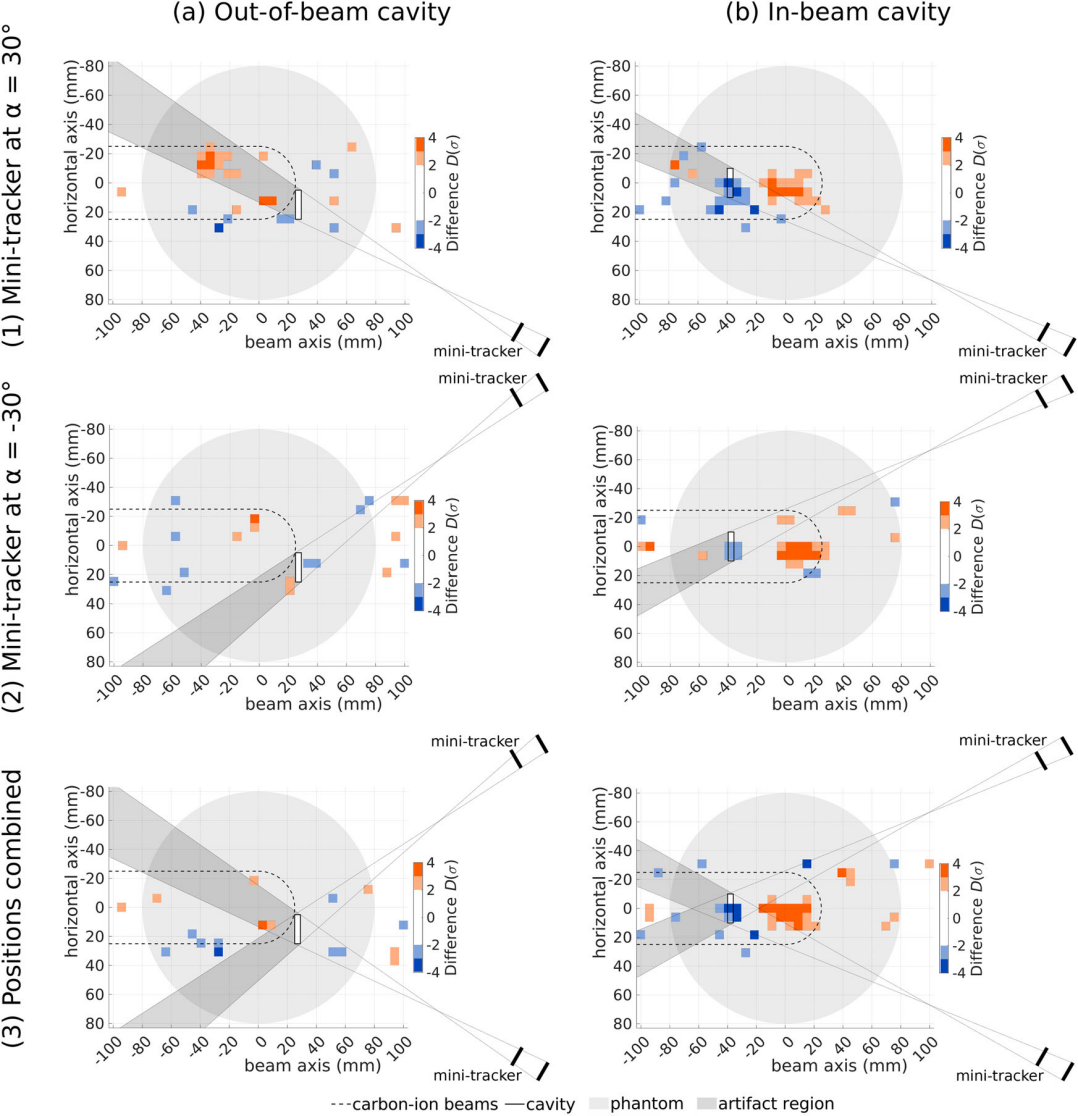
Difference in units of standard deviation D(σ) of measured FV distributions relative to their reference. The two columns represent the different air‐cavity positions: (a) out‐of‐beam cavity and (b) in‐beam cavity. Rows (1) and (2) show data from two different mini‐tracker positions, which are combined in row (3). The phantom is shown in light gray, the region accessed by primary carbon ions is delineated with a dashed line and the white air cavities are outlined with a solid line. To facilitate the interpretation of the data, the area where fragments are produced that cross the cavity as well as both layers of the mini‐tracker is shaded in dark gray. In this region artifacts can potentially occur.

**FIGURE 5:**
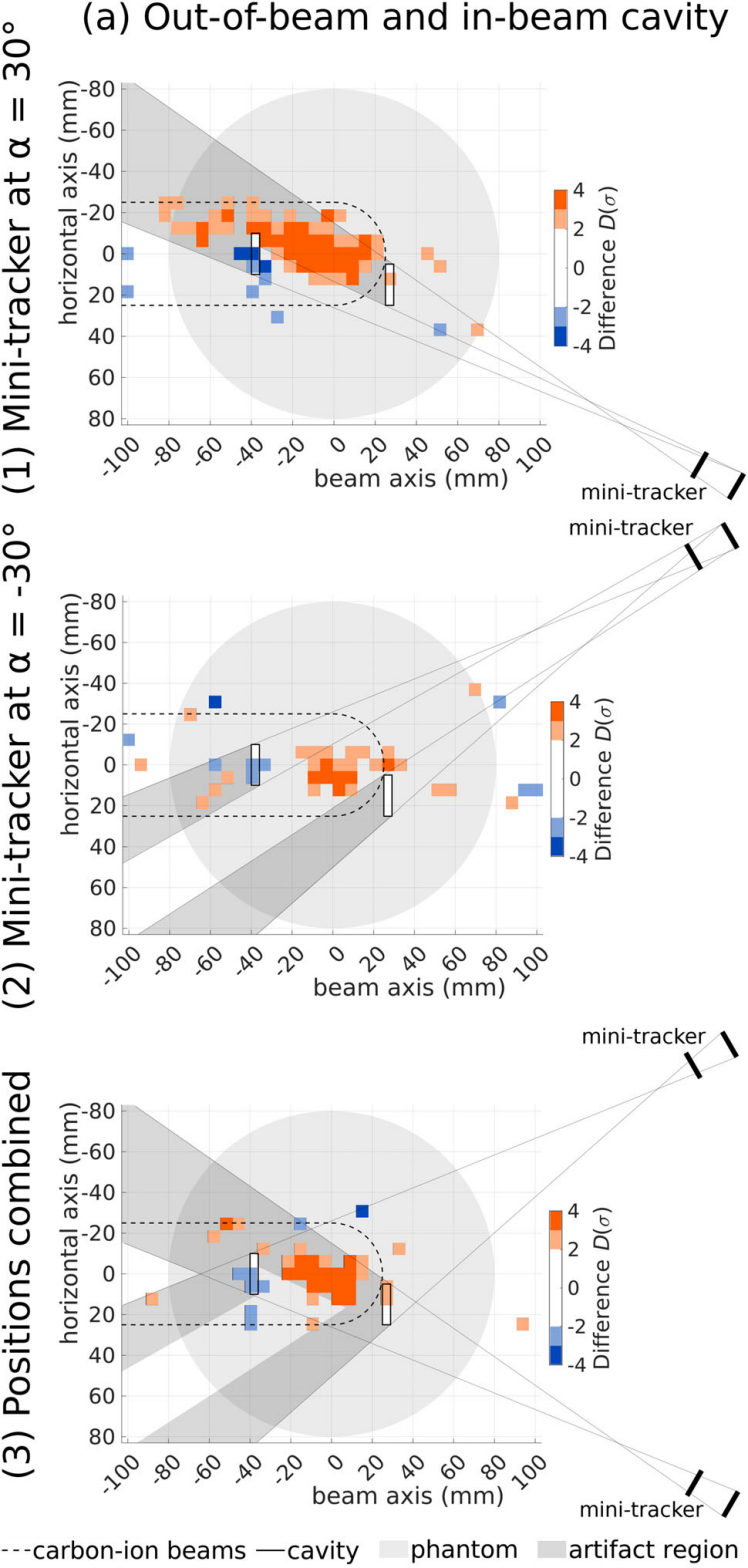
Difference in units of standard deviation D(σ) of measured FV distributions with both the out‐of‐beam and the in‐beam cavity relative to their reference. Rows (1) and (2) show data from two different mini‐tracker positions, which are combined in row (3). The phantom is shown in light gray, the region accessed by primary carbon ions is delineated with a dashed line and the white air cavities are outlined with a solid line. To facilitate the interpretation of the data, the area where fragments are produced that cross the cavity as well as both layers of the mini‐tracker is shaded in dark gray. In this region artifacts can potentially occur.

Figures 4 and 5 show the results of measurements with an air cavity outside of the region accessed by carbon‐ion beams (out‐of‐beam cavity, Figure 4 column (a)) and in the region accessed by carbon‐ion beams (in‐beam cavity, Figure 4 column (b)) as well as with both cavities inserted simultaneously (Figure 5). Four repetitions with the mini‐tracker at $\alpha =30^{\circ }$ are used for row (1) and four repetitions with the mini‐tracker at $\alpha =-30^{\circ }$ are shown in row (2). For row (3), two repetitions of each mini‐tracker position are used, both for the reference and the measurement with air cavities. This ensures the same fragment count for all investigated scenarios.

To facilitate the interpretation of the plots, additional structures are depicted. The position of the air cavity is outlined and the mini‐tracker is sketched. Artifacts from absorption and scattering effects of a density change are expected in a specific region, which is here shaded in dark gray. Fragments from this region cross the air cavity and both layers of the mini‐tracker, where they are detected and included in the analysis. Their scattering and absorption probability is altered by the cavity, which potentially produces an artifact. This region is constructed with the depicted auxiliary lines and is called *artifact region* in the following.

Figure 4 (1a) shows a clear signal produced by the out‐of‐beam cavity. There is an increase in the number of FVs of up to 4σ within the artifact region, which can be explained by the decreased absorption and scattering probability for those fragments. The deviations outside of the artifact region are consistent with statistical fluctuations.

The in‐beam cavity also produces a signal as can be seen in Figure 4 (1b), although with different characteristics. The number of tracked fragments is decreased in the region around the cavity by up to 3.5σ and increased downstream by more than 4σ. This signal originates from the influence of the cavity on the primary ions. Almost no nuclear interactions occur within the 4 mm‐thick air cavity, resulting in almost no fragments being produced there. Due to the tracking resolution and because the cavity does not extend across the entire lateral extension of the treatment plan, the signal takes the shape of a small but significant decrease around the cavity. The increase downstream of the cavity is due to a range overshoot of the primary ions. There is no significant difference in the artifact region and thus no signal from the influence of the in‐beam cavity on fragment scattering and absorption.

Figure 4 Row (2) shows the same measurements but with the mini‐tracker at $\alpha =-30^{\circ }$. As expected, there is no signal from the out‐of‐beam cavity whereas that of the in‐beam cavity is still present. This behavior can be explained by the underlying effects producing the signals. In the case of the out‐of‐beam cavity the signal stems from an influence on the measured fragments, which depends on the location of the cavity and the mini‐tracker. In contrast to this, the influence of the in‐beam cavity on the primary ions depends on the cavity location as well as the beam direction and is thus independent of the mini‐tracker position in a first approximation. The reduced magnitude of the in‐beam‐cavity signal in Figure 4(2b) could be due to a contending fluctuation in the measured distributions. Potential sources of such fluctuations are mentioned in Section 3.1.

The combination of data from the two mini‐tracker positions in Figure 4 row (3) suppresses the artifact of the out‐of‐beam cavity such that it cannot be distinguished from statistical fluctuations. This is due to the artifact from Figure 4(1a) being present in only two of the four repetitions used in Figure 4(3a), which reduces its statistical significance to below 2σ. Meanwhile, the signal of the in‐beam cavity remains unchanged by the combination of two mini‐tracker positions with respect to amplitude and shape, as the signal is very similar for both mini‐tracker positions.

Figure 5 shows the results obtained with both air cavities in the phantom. Row (1) shows a decrease in the number of FVs at the in‐beam cavity of up to 4σ and an increase in the artifact region, which is especially strong downstream of the in‐beam cavity, where it reaches 7σ. In the case of the mini‐tracker at $\alpha =-30^{\circ }$ in row (2), a signal similar to that of the in‐beam cavity in Figure 4 column (b) is visible. However, in the artifact regions, no signal is present. This demonstrates that the effects of the two cavities are additive in a first approximation.

When the two mini‐tracker positions are combined in Figure 5 row (3), the signal again looks similar to that of the in‐beam cavity only, except for a slight increase of the signal in the artifact region corresponding to the mini‐tracker at $\alpha =30^{\circ }$. However, this increase is suppressed compared to Figure 5 row (1), while the effect from the increased range of the primary ions is maintained.

## 4 DISCUSSION

This work shows that a clinically irrelevant change outside of the region accessed by carbon‐ion beams can lead to a significant artifact in monitoring with charged nuclear fragments. For the envisaged application of the method to suggest control CTs for patients when and only when they are needed, the occurrence of artifacts needs to be minimized. Thus, the identification and suppression of artifacts from out‐of‐beam changes is of utmost importance.

It was shown that the artifact resulting from a change in the absorption and scattering of fragments differs from the signal resulting from a change in the production of fragments. The most promising approach for differentiation is the dependence of the artifact on the mini‐tracker position. If several mini‐trackers are arranged at different positions around the patient, artifacts from out‐of‐beam changes will appear at different positions in the FV distribution, whereas in‐beam changes produce signals in the same location for each mini‐tracker position. Based on this, the signals can be differentiated visually and clinically irrelevant density changes may be identified. Additionally, this work introduces an efficient method to suppress absorption and scattering artifacts by combining the data from the different mini‐trackers before analysis. This reduces the amplitude of artifacts relative to statistical fluctuations as they are combined with data that does not exhibit an artifact in the same region. At the same time, clinically relevant signals are preserved, as they occur in the same region and add up. These findings are implemented in the design of the detection system for our ongoing clinical study, which consists of seven mini‐trackers at different positions around the patient with the largest angle between two mini‐trackers being $90^{\circ }$.^10^

This work presents a simplified case where the in‐beam cavity signal is dominated by fragment production effects while fragment scattering and absorption lead to the out‐of‐beam artifact. Therefore, here the differentiation between production as opposed to scattering and absorption effects is equivalent to a differentiation between the two types of density changes. However, in‐beam changes may also produce significant scattering and absorption signals, if they are larger in size or located deeper inside the patient. As they also influence the production of fragments, the method found in this work can still be used to identify them as clinically relevant changes. However, in these cases the scattering and absorption signal holds valuable information, for example on the location of the in‐beam change. This information is encoded differently in the scattering and absorption signal than in the production signal. Therefore, it might be useful to split the signal of an in‐beam change into the respective contributions originating from fragment scattering and absorption on the one hand and fragment production on the other hand. This can be achieved by using the findings of this work and separate the part of the signal that changes with the mini‐tracker position from the part that is unaltered. After separation, both parts of the signal could be analyzed with respect to the location of the change using different analysis methods optimized for scattering and absorption or production effects. This could potentially improve the localization in depth, which has been found to be especially challenging for deep‐seated density changes.^7^

## 5 SUMMARY AND CONCLUSION

This work investigates artifacts in carbon‐ion radiotherapy monitoring using charged nuclear fragments, which can arise from anatomical changes outside of the region accessed by carbon‐ion beams. As such, changes that do not alter the dose distribution in the target volume are deemed clinically irrelevant. This manuscript studies the characteristics of such artifacts and compares them to clinically relevant signals from anatomical changes in the region accessed by carbon‐ion beams. The aim is to develop strategies for the identification and suppression of out‐of‐beam artifacts, to prevent their misinterpretation as clinically relevant signals in a future clinical application of this monitoring method.

In this work, anatomical changes inside and outside of the carbon‐ion beams were generated by inserting air cavities at different positions in a homogeneous head‐sized PMMA cylinder. This head phantom was irradiated with a clinically realistic treatment plan. During irradiation, charged nuclear fragments arising from nuclear interactions of the primary carbon ions where tracked with a mini‐tracker based on semiconductor detectors. The measured tracks allow the reconstruction of the approximate origins of the fragments, resulting in a fragmentation vertex distribution in the phantom as a basis of the analysis. FV distributions measured with air cavities in the phantom were compared to such without changes to the phantom. This was done for two different positions of the mini‐tracker.

The analysis revealed differences between relevant signals and artifacts due to the different underlying effects. The artifact of the out‐of‐beam cavity arises from a reduction in the absorption and scattering of fragments that cross the cavity on their way to the mini‐tracker. This explains the observed dependence of the artifact on the mini‐tracker position. In contrast, the relevant signal of the in‐beam cavity is independent of the mini‐tracker position in a first approximation, as it results from a change in the range of the primary carbon ions. This observation led to the strategy of suppressing the artifact by combining data from different mini‐tracker positions. It could be demonstrated that combining the FV distribution from both mini‐tracker positions without changing the overall amount of data led to a suppression of the out‐of‐beam artifact below 2 σ while retaining the relevant signal of the in‐beam cavity. This effect was also seen for the measurements with both cavities in the phantom simultaneously. These findings motivate the design of the detection system used in our ongoing clinical trial, where seven mini‐trackers are arranged at different positions around the patient.^10^

This investigation demonstrates the feasibility of identifying and suppressing artifacts that originate from anatomical changes outside of the primary beams in carbon‐ion radiotherapy monitoring. Central to this approach is the combination of tracking data from different observation angles around the patient. The clinical potential of the proposed identification and suppression method for more complex scenarios will have to be tested with patient data and verified in Monte Carlo simulations.

## CONFLICT OF INTEREST STATEMENT

The authors declare no conflicts of interest.
